# Infant frontal alpha asymmetry predicts social attention and transdiagnostic risk for emotional reactivity

**DOI:** 10.1002/jcv2.70089

**Published:** 2026-01-12

**Authors:** Viviane Valdes, Niccola Lutri, Haerin Chung, Charles A. Nelson

**Affiliations:** ^1^ Boston Children's Hospital (Division of Developmental Medicine) Boston Massachusetts USA; ^2^ Harvard Medical School (Department of Pediatrics) Boston Massachusetts USA; ^3^ Northeastern University (Bouvé College of Health Sciences) Boston Massachusetts USA; ^4^ Harvard Graduate School of Education Boston Massachusetts USA

**Keywords:** ADHD, ASD, depression, emotional reactivity, frontal alpha asymmetry, social attention

## Abstract

**Background:**

Differences in Frontal Alpha Asymmetry (FAA), derived from the electroencephalogram (EEG), have been associated with approach‐withdrawal behavior, although inconsistently. The current study examined how early patterns of FAA during the first 2 years of life relate to various socioemotional characteristics (at 2 years) and ultimately psychopathology symptoms (at 3 years).

**Methods:**

Using a longitudinal prospective cohort design, 211 families were followed during the first two years of life. EEG data were obtained at 2‐, 6‐, 9‐, 12‐, and 24 months. Child socioemotional characteristics were assessed at 2 years using the Infant‐Toddler Social and Emotional Assessment. Child psychopathology symptoms were assessed at 3 years using the Child Behavior Checklist (CBCL). Structural Equation Models were used for analyses.

**Results:**

Greater relative left frontal asymmetry at 12 months was associated with greater social attention (*β* = 0.39, *p* = 0.0006), empathy (*β* = 0.31, *p* = 0.01), and social approach (*β* = 0.33, *p* = 0.0038) at 2 years. FAA was not significantly associated with inhibition to novelty (i.e., withdrawal), although it did covary with other socioemotional characteristics. Only higher levels of social attention were significantly associated with subsequent psychopathology symptoms, specifically with lower levels of emotional reactivity (*β* = −0.29, *p* = 0.012). Social attention also predicted categorical symptoms including Attention‐Deficit/Hyperactivity Disorder, Autism Spectrum Disorders, and Depression.

**Conclusion:**

FAA lateralization at 12 months was linked to early social attention, empathy, and social approach at 2 years. However, only lower levels of social attention predicted higher emotional reactivity at 3 years. These findings highlight the importance of attentional processes (e.g., social attention), rather than behavioral differences (e.g., approach/withdrawal) for the link between FAA and psychopathology. Future research replicating these findings and on interventions addressing underlying attentional/sensory processing differences may be relevant to support the development of emotion regulation.

## Key Points

**What's known?**

- Frontal alpha asymmetry (FAA) has been linked to approach‐withdrawal motivation in past work, but observed associations have not always been consistent.

**What's new?**

- In a longitudinal cohort, we found that greater relative left FAA at **12 months** predicted higher **social attention**, **empathy**, and **social approach** at **2 years**, but not inhibition to novelty.
- Higher levels of social attention in particular mediated paths to **lower emotional reactivity** at **3 years,** and were also associated with lower levels of **ADHD**, **Autism**, and **Depression** symptoms.

**What's relevant?**

- These findings emphasize the role of attention and social‐information processing as a pathway linking FAA to transdiagnostic psychopathology risk in early childhood.

## INTRODUCTION

The differences between left and right frontal hemispheric activity in the alpha frequency range during recordings of electroencephalography (EEG) have been referred to in the literature as “frontal alpha asymmetry” (FAA). Researchers have sought to examine FAA and their behavioral correlates to understand what these patterns of brain activation may be indexing (Ahern & Schwartz, 1985; Davidson et al., 1985, 1990; Tucker, 1981). Largely relying on the approach‐withdrawal hypothesis (Henriques & Davidson, 1991), greater relative left FAA (i.e., decreased activation of the right frontal lobe when compared to the left; hereafter referred to as left frontal asymmetry (LFA)) is associated with the appetitive motivational system toward the perceived stimulus and other potential positive emotions (e.g., states of calm) (Henriques & Davidson, 1991). In contrast, greater relative right FAA (the opposite pattern of activation; hereafter referred to as RFA) is associated with withdrawal behaviors and negative emotions (e.g., feelings of fear or sadness) relative to the perceived stimulus (Henriques & Davidson, 1991).

Consequently, FAA has been predominantly studied in relation to behavioral inhibition systems (BIS), behavioral approach systems (BAS), and more recently with empathic responses. Beginning with BIS and BAS, FAA has been proposed as a component of core neural systems that facilitate motivational and affective responses for threat avoidance (BIS) or appetitive behaviors (BAS) (Angus & Harmon‐Jones, 2016; Gray & McNaughton, 2003; Harmon‐Jones & Gable, 2018). Patterns of LFA have been consistently associated with behavioral approach responses; however, literature on behavioral inhibition has been less consistent with some studies finding null associations and some finding associations between RFA and behavioral inhibition (Angus & Harmon‐Jones, 2016; Carver & White, 1994; Coan & Allen, 2003; Harmon‐Jones & Allen, 1997; Harmon‐Jones & Gable, 2018; Quaedflieg et al., 2015; Sutton & Davidson, 1997).

It has proved challenging to determine why behavioral inhibition is inconsistently associated with RFA. One possibility is that these inconsistencies may be a product of individual differences in cognitive and affective processes (e.g., psychological factors like avoidance strategies) that are not observable at the behavioral level (Angus & Harmon‐Jones, 2016; Harmon‐Jones & Gable, 2018). e.g., it is possible that some individuals may appear socially engaged at a behavioral level, while internally they still rely on avoidance strategies (e.g., redirecting their attention) to manage perceived threat sensitivities. FAA may be more related to some of these internal processes that are less directly observable than to overt processes like behavioral inhibition. With regards to approach‐related behavior, in addition to consistent associations with LFA, some work has also examined approach‐related behaviors by specific forms/domains. For instance, studies have found links between LFA and self‐reported sensation‐seeking (i.e., the desire to seek novel and intense sensations as well as the willingness to take risks in order to obtain them) (Harmon‐Jones & Gable, 2018; Santesso et al., 2008). Other research has also found that individuals that have greater relative LFA are also more willing to invest higher levels of effort in pursuing rewards, even when the likelihood of obtaining the reward has a low likelihood (Harmon‐Jones & Gable, 2018; Hughes et al., 2015).

Given the possibility that internal processes may be more consistently linked to FAA and drive motivational responses like behavioral inhibition and approach, researchers have begun to examine associations between FAA and traits related to empathic processing as a possible explanatory link that may drive motivational responses. In other words, that it may actually be differences in levels of empathic processing and understanding that could drive avoidance or approach behaviors, possibly explaining patterns of RFA/LFA more consistently. For instance, research in adolescent boys has found that callous‐unemotional traits tend to be associated with RFA (Batky et al., 2020) and with lower levels of empathy in preschool‐aged children of depressed mothers (Jones et al., 2000). Greater relative LFA has been associated with a tendency toward altercentric perspectives (i.e., taking an observed agent's perspective in order to understand and process their intentions) (Zappullo et al., 2024). Greater relative LFA has also been observed when individuals report higher levels of social motivation and responsiveness (Soriano et al., 2020).

FAA has also been studied to examine risk for a variety of psychopathology outcomes (Allen et al., 2018). In existing work depressive disorders have been the most studied, with documented associations between depressive symptoms (e.g., dysphoria, lassitude) and greater relative RFA (Nelson et al., 2018). Importantly, it appears that only certain aspects of depressive disorders are associated with patterns of RFA given symptom heterogeneity across individuals meeting criteria for depressive disorders (Allen et al., 2018; Coan et al., 2006). For instance, only individuals with depression onset in childhood and without comorbid anxiety disorders display this pattern (greater relative RFA) of activation (Nusslock et al., 2018). Adults with a history of depression and comorbid symptoms of anxiety (i.e., anxious apprehension such as that observed in generalized anxiety disorder, obsessive‐compulsive disorder, separation anxiety disorder) did not differ from controls in patterns of laterization for FAA.

While FAA has been traditionally understood through the approach‐withdrawal framework and consequently studied in relation to anxiety/depressive symptoms, emerging evidence also links FAA to individual differences in internal psychological processes (e.g., social attention, empathy, perspective‐taking) (Batky et al., 2020; Jones et al., 2000; Soriano et al., 2020; Zappullo et al., 2024). Given the prominent role of attentional and social differences in Attention‐Deficit/Hyperactivity Disorder (ADHD) and Autism Spectrum Disorders (ASD), researchers have examined how these disorders may relate to FAA. Findings from research examining associations between FAA and ADHD specifically has been mixed in terms of patterns of lateral activation. Some research has found evidence for greater RFA across the lifespan (Baving et al., 1999; Hale et al., 2009, 2010; Keune et al., 2011, 2012), while other research findings greater relative LFA (Baving et al., 1999; Jaworska et al., 2013), or no differences in patterns of activation for FAA between children with ADHD and controls (Gordon et al., 2010). More recent work suggests that these differences may be attributable to some of the heterogeneity in emotional responses for individuals with ADHD, with negative affective symptoms most associated with patterns of greater relative RFA among individuals with ADHD (Alperin et al., 2019).

Finally, some work has also examined associations between patterns of activation in FAA ASD. Research among infants with an elevated risk for developing autism (i.e., siblings of children with an ASD diagnosis) suggests that by 18 months, those at increased risk for ASD have greater relative LFA (Gabard‐Durnam et al., 2015). Findings across studies at older ages (8+ years) suggests that those with ASD tend to have relatively stronger LFA compared to peers without ASD (Schiltz et al., 2018; Sutton et al., 2005). However, other work finds null and/or inconsistent associations (Burnette et al., 2011; Neuhaus et al., 2023). Importantly, recent work suggests that it may be the case that specific symptoms in ASD (e.g., eye contact, reporting events to others, and engaging in reciprocal social communication) tend to be linked with FAA (Bitsika et al., 2024). Transdiagnostic frameworks, such as the Research Domain Criteria (RDoC) serve as a useful lens to understand why FAA may relate a wide variety of categorical disorder outcomes. Inconsistent findings across categorical domains also point to the need to study psychopathology outcomes transdiagnostically to better understand the mechanistic processes involved.

To summarize, existing research has most consistently found associations between greater LFA and approach motivation, while associations with RFA and behavioral inhibition have been more inconsistent in existing research. A relatively smaller body of work has found links between RFA and lower levels of empathy; LFA has been linked to altercentric perspectives (i.e., taking on the agent's perspective to understand their intentions) and greater social motivation/responsiveness. Furthermore, FAA has been examined at a diagnostic‐level with various forms of psychopathology and neurodevelopmental conditions (e.g., depression, anxiety, ADHD, ASD) but findings have also been inconsistent, with some emerging work pointing toward the possibility that lateralized patterns of FAA could be linked with specific transdiagnostic symptoms as opposed to disorders. Because the existing literature on FAA and psychopathology has focused on categorical disorders, it has often disproportionately studied older individuals (e.g., adolescents, adults, and to a lesser extent school‐aged children), with limited attention to early childhood.

Despite the breadth of existing literature on FAA, several gaps remain in the literature. For one, while the field has long accepted FAA as a marker of approach and withdrawal motivation, inconsistent findings point to the possibility that FAA may be indexing other processes that co‐occur with approach/withdrawal (e.g., social information processing like altercentric perspective‐taking, eye contact, event reporting, reciprocal social communication, or other forms of empathic responding) (Bitsika et al., 2024; Zappullo et al., 2024). In line with this, meta‐analytic and review findings caution that the observed effects that have been documented are often small and inconsistent (Allen et al., 2018; Kolodziej et al., 2021; van der Vinne et al., 2017). Research focusing on transdiagnostic features is also needed to reconcile inconsistencies in existing work relying on diagnostic categories that are often heterogenous. Finally, research with longitudinal samples in early childhood is also needed, as the first 2 years of life are characterized by rapid neurobiological development and this period remains relatively understudied in the FAA‐psychopathology literature (Feldman, 2015; Nelson & Gabard‐Durnam, 2020).

To address these gaps, the aim of the current study was to examine how early patterns of FAA (measured longitudinally at 2, 6, 9, 12, and 24 months) are linked with various socioemotional characteristics (measured at 2 years of age), and with multiple transdiagnostic and categorical psychopathology symptoms at age 3, through potential mediational pathways. Given findings from existing research suggesting that FAA is most consistently associated with approach‐related social processing and behaviors, we hypothesized a priori that greater relative LFA would predict higher social attention, empathy, and social approach at 2 years of age, with weaker or null associations to inhibitory control. Additionally, we sought to model patterns of covariance among mechanisms and outcomes (e.g., such as associations between inhibition to novelty and social approach, or between transdiagnostic and categorical psychopathology symptoms), even in the absence of direct associations with FAA. To inform model fitting, exploratory associations between FAA at various developmental time points (2, 6, 9, 12, and 24 months) and socioemotional characteristics (defined a priori), and between socioemotional characteristics and psychopathology domains were also examined.

## METHODS

### Procedure

Participants were recruited during regularly scheduled pediatric visits at Boston Children’s Hospital Primary Care Center (CHPCC) in Boston, Massachusetts. The first two groups of children were enrolled at 2 months of age, and the third group was enrolled at 24 months of age. Families in the current analyses are part of a longitudinal study originally designed to examine developmental responses to early adversity. Exclusion criteria included gestational age <36 weeks, birth weight <2.5 kg (20th percentile), identified genetic, metabolic, syndromic, or progressive neurological disorder (including epilepsy, Down Syndrome, Rett Syndrome, Tuberous Sclerosis, Neurofibromatosis, Fragile X Syndrome), uncorrected vision difficulties, or prenatal, postnatal, or birth‐related complications (i.e., extended stay on the NICU). After enrollment, families were followed through their child's 36‐month study visit. Study procedures were approved by the Institutional Review Board at Boston Children's Hospital, and parents or legal guardians provided written informed consent prior to the initiation of study activities. The first group of children recruited included 59 mother‐child dyads from CHPCC; recruitment took place from January 2016 to May 2017, and data was collected between February 2016 and November 2020. The second group of children recruited included 44 mother‐child dyads from CHPCC; recruitment took place from January 2019 to November 2021, and data took place from February 2019 to December 2024. The third group of children recruited included 108 mother‐child dyads from CHPCC; recruitment took place from November 2022 to November 2023, and data collection took place from November 2022 to December 2024. To be included in the current analyses, participants had to provide data for at least one longitudinal time point of baseline EEG at 2 months (*n* = 90), 6 months (*n* = 77), 9 months (*n* = 71), 12 months (*n* = 72), or 24 months (*n* = 106). The final sample recruited was comprised of families that are historically underrepresented in child development research, with 44.50% of families identifying as racially Black/African American and 29.86% as Hispanic/Latine. Families were also predominantly from low and middle‐income households, with 45.5% of the sample reporting income below $75,000 (Celestin et al., 2025; Valdes et al., 2020).

### Measures

#### Baseline EEG

Infant recordings of resting‐state EEG were completed using a 128‐channel Hydrocel Sensor Net System (EGI Inc). Recordings took place in dimly lit environments with a low‐electrical‐signal‐background. Infants were positioned 60 cm from a computer monitor (Dell model P2314Ht, 49 × 29 cm), seated and facing forward on their parent's lap (or, at 2 months, held over the shoulder by their parent who was seated backwards). EEG data were recorded using the NetAmps 300 Amplifier (NetStation, version 4.5.4), or the NetAmps 400 Amplifier (NetStation, version 5). The data were amplified and sampled at 500 Hz while infants viewed a non‐task related video featuring infant toys for up to 5 min (mean impedance, <100 kΩ). A trained researcher interacted minimally with the infants, using a toy and/or tapping on the back of the screen to redirect their attention to the screen only when necessary.

#### EEG analysis

Raw EEG files were exported from NetStation, version 4.5.4 in MATLAB format (MathWorks Inc) and preprocessed using the Harvard Automated Processing Pipeline for Electroencephalography, which is optimized for infant EEG and utilized with MATLAB, version 2014b and EEGLAB, version 14.0.0b. Within the Harvard Automated Processing Pipeline for Electroencephalography, data were down‐sampled to 250 Hz and band‐pass filtered between 1 and 100 Hz. Rim electrodes (E1, E107, E113, E114, E119, E120, E121, E125, E126, E127, E128, E14, E17, E21, E25, E32, E38, E43, E44, E48, E49, E56, E63, E68, E73, E8, E81, E88, E94, E99) were excluded as artifact is common in these changes in infant recording. Wavelet thresholding was applied to correct artifacts, and 2‐s segments were extracted. Segments that still contained artifacts were removed before re‐referencing to the average. Bad channels were interpolated. Five EEGs were excluded for having fewer than 20 segments (40 s of total EEG) post processing, and 9 EEGs were excluded for having low signal‐to‐noise (pre/post‐wavelet correlations of <0.1) All recordings had three or fewer bad channels in frontal regions. Out of 416 EEG observations across all longitudinal time points, 402 EEG observations were included in our analyses.

Power spectra for each channel were obtained using a fast Fourier transform with a multitaper window. For each participant, mean power across all segments was computed. Log10‐transformed absolute power was calculated for each frequency band of interest: *Δ* (2–4 Hz), *θ* (4–6 Hz), low *α* (6–9 Hz), high *α* (9–13 Hz), *β* (13–30 Hz), and *γ* (30–50 Hz). Given the aims of the current study, alpha values were retained for statistical analysis. These values were then averaged across selected electrodes (detailed below). The current study defined alpha using the low *α* (6–9 Hz) band, in line with recommendations from Vincent et al. (2021).

#### Frontal alpha asymmetry calculation

**FIGURE 1:**
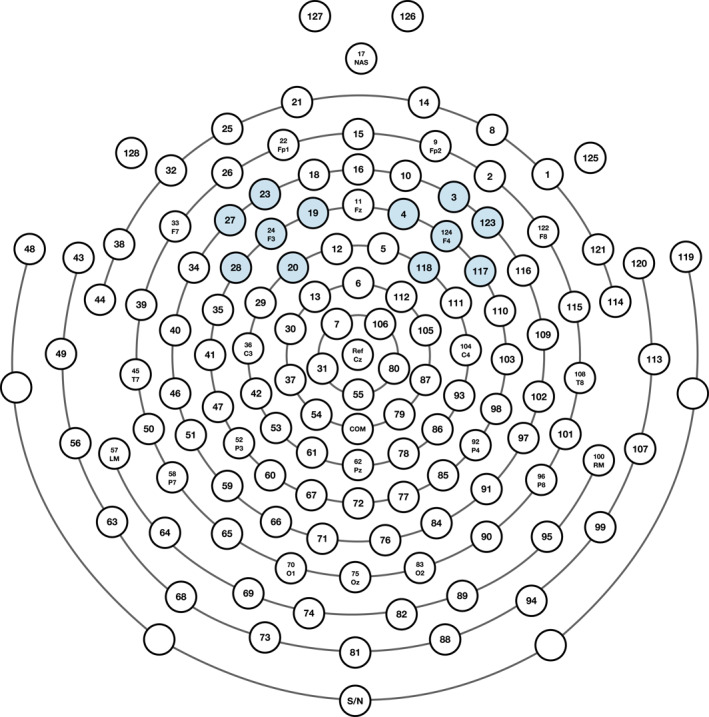
Map of HydroCel geodesic sensor net 128‐region layout with frontal electrodes marked in blue.

For the current analyses, FAA was derived using the procedure recommended by Vincent et al. (2021) which demonstrated the most stability in longitudinal study designs. After relative alpha power was calculated and log‐transformed (calculated as a percentage of power in the alpha band divided by total power in all frequency bands), relative alpha values for average electrodes surrounding the F3 (19, 20, 23, 24, 27, and 28) and F4 (3, 4, 117, 118, 123, and 124) 10−20 positions were subtracted to derive FAA (Figure 1). Negative asymmetry values are indicative of right frontal lateralization (i.e., greater activation in right frontal alpha) and positive asymmetry values are indicative of left frontal lateralization (i.e., greater activation in left frontal alpha). In line with work by Vincent et al. (2021), FAA estimates in the current sample were also found to be reliable longitudinally from infancy through 24 months (Cronbach's *α* = 0.73).

#### Child socioemotional characteristics

Child socioemotional characteristics were assessed at 2 years of age using the Infant‐Toddler Social and Emotional Assessment (ITSEA) (Carter et al., 2003). The ITSEA is a parent‐report questionnaire that assesses socioemotional problems and competencies of children 12–35 months and 30 days. The ITSEA provides composite scores across four broad domains: Externalizing, Internalizing, Dysregulation, and Competence. The respondent rates individual items on a three‐point scale (0 indicating “Not True/Rarely,” 1 indicating “Somewhat True/Sometimes,” 2 indicating “Very True/Often”) that are then summed to calculate raw scores. T‐scores are calculated from norms based on age and sex and have a mean of 50 and a standard deviation of 10. For the current analyses, scales within the ITSEA Externalizing, Internalizing, Dysregulation, and Competency domains were used (Carter et al., 2003). Specifically, social attention, empathy, social approach, and inhibition to novelty were selected for socioemotional characteristics given evidence in existing research. The Externalizing domain includes the Activity/Impulsivity, Aggression/Defiance, and Peer Aggression scales (Carter et al., 2003). The Internalizing domain includes the Depression/Withdrawal, General Anxiety, Separation Distress, and Inhibition to Novelty scales (Carter et al., 2003). The Dysregulation domain includes the Sleep, Negative Emotionality, Eating, and Sensory Sensitivity Scales (Carter et al., 2003). The Competency domain includes Compliance, Attention, Imitation/Play, Mastery Motivation, Empathy, and Prosocial Peer Relations scales.

#### Child psychopathology symptoms

Child psychopathology symptoms were assessed at 3 years of age using the parent‐report Child Behavior Checklist (CBCL) for ages 1.5–5 years (Achenbach & Edelbrock, 1991; Achenbach & Rescorla, 2000). The parent‐report version obtains parent ratings for 99 problem items that can be scored according to syndrome or DSM‐oriented scales (Achenbach et al., 2001). Parents were asked to rate how true each item was for their child in the prior 6 months on a three‐point scale (“not true” = 0, “somewhat or sometimes true” = 1, “very true or often true” = 2). T‐scores on the CBCL have a mean of 50 and a standard deviation of 10. Scores in the 60–63 range are considered borderline for psychopathology/neurodevelopmental conditions, and scores greater than 63 are considered clinically elevated. For the current analyses, both the syndrome and DSM‐oriented scales were used. Syndrome scales on the CBCL include Emotionally Reactive, Anxious/Depressed, Somatic Complaints, Withdrawn, Sleep Problems, Attention Problems, and Aggressive Behaviors (Achenbach et al., 2001). The DSM‐oriented scales on the CBCL include Depressive Problems, Anxiety Problems, Autism Spectrum Problems, Attention‐Deficit/Hyperactivity Problems, and Oppositional Defiant Problems.

### Statistical analysis

Statistical analysis was carried out using Stata SE Version 17 (College Station, TX: StataCorp LLC). Descriptive statistics for sociodemographic variables were calculated to characterize the sample. Effects with a *p*‐value of less than 0.05 (two‐tailed) were considered statistically significant. In order to address the proposed aims, a Structural Equation Modeling (SEM) approach was used. Preliminary analyses tested pairwise comparisons with FAA assessed at each longitudinal time point (2, 6, 9, 12, and 24 months of age), multiple socioemotional characteristics (using selected scales from the ITSEA at 2 years of age; defined a priori), and with multiple psychopathology transdiagnostic domains and categorical disorder symptoms (using the CBCL at 3 years of age). Statistical significance testing for preliminary analyses is reported both unadjusted and with adjustments for multiple comparison using the Benjamini–Hochberg False Discovery Rate (FDR) procedure. Subsequent model building was informed by the strongest and most consistent effect sizes observed in these preliminary analyses. In addition to testing direct and indirect associations with FAA, we modeled covariation among socioemotional and psychopathology symptoms, even in the absence of direct links/paths with FAA. All models were estimated using the maximum likelihood method. A priori paths to social attention, empathy, social approach, and inhibition to novelty were selected for socioemotional characteristics given evidence in existing research. Model fit was evaluated using multiple fit indices including the chi‐square (*χ* ^2^) goodness‐of‐fit test, root mean square error of approximation (RMSEA), and comparative fit indices (i.e., Akaike's information criterion (AIC), Bayesian information criterion (BIC), Comparative Fit Index (CFI), Tucker–Lewis Index (TLI)).

## RESULTS

### Sample characteristics

Descriptive statistics for the sample's demographic characteristics are presented in Table 1.

**TABLE 1:** Descriptive statistics for sample characteristics (*N* = 211).

Variables	*n*	%
Child sex		
Male	98	46.45
Female	101	47.87
Not reported	12	5.69
Child race		
White	38	18.0
Black/African American	94	44.5
Asian American, Native American, or Pacific Islander	15	7.1
Other	17	8.1
Not reported	47	22.3
Child ethnicity		
Non‐Hispanic/Latine	82	38.86
Hispanic/Latine	63	29.86
Not reported	66	31.28
Home language		
English	102	48.34
Spanish	11	5.21
Both English and Spanish	14	6.64
Other	20	9.48
Not reported	64	30.33

### Preliminary analyses

Preliminary pairwise comparisons used to inform model fitting in Structural Equation Models are presented in Tables 2 and 3. Associations between FAA and social attention were significant at 9 months before correction for multiple comparison (*r* = 0.315, *p* = 0.041), but effects were stronger at 12 months (*r* = 0.360, *p* = 0.016). Associations between FAA and other socioemotional areas like empathy (*r* = 0.336, *p* = 0.028) and social approach (*r* = 0.299, *p* = 0.048) were strongest at 12 months (Table 2). Effects between FAA at 2 months, 6 months, and 24 months and each of the selected socioemotional characteristics were all null, even without adjustments for multiple comparisons (Table 2). Only associations between FAA at 12 months and social attention, empathy, and social approach at 2 years survived corrections for multiple comparisons. Given that these preliminary findings point to the most consistent effects between FAA at 12 months and multiple socioemotional characteristics at 2 years, subsequent SEM models focused on these. Associations between socioemotional characteristics at 2 years and psychopathology symptoms/neurodevelopmental conditions at 3 years are presented in Table 3. Only associations between social attention at 2 years and transdiagnostic/categorical symptoms at 3 years survived corrections for multiple comparisons, and these paths were included in subsequent SEM models.

**TABLE 2:** Pair‐wise correlations between all longitudinal timepoints of FAA and selected socioemotional characteristics.

Variables	FAA (2 mo)	FAA (6 mo)	FAA (9 mo)	FAA (12 mo)	FAA (24 mo)
Social attention (2 yr)	−0.101 (0.520)	0.122 (0.472)	0.320 (0.041)	0.360* (0.016)	−0.065 (0.588)
Empathy (2 yr)	−0.038	0.149	0.315	0.336*	−0.160
	(0.809)	(0.385)	(0.048)	(0.028)	(0.187)
Social approach (2 yr)	−0.003	−0.143	0.174	0.299*	−0.153
	(0.986)	(0.399)	(0.275)	(0.048)	(0.200)
Inhibition to novelty (2 yr)	−0.204	−0.151	0.360	0.261	−0.066
	(0.202)	(0.380)	(0.024)	(0.095)	(0.592)

*Note*: Exact effect sizes (first row) and *p*‐values (second row) are reported for each pairwise association. Associations that survive Benjamini–Hochberg FDR correction at *q* = 0.05 are marked with an asterisk.

Abbreviations: FAA, frontal alpha asymmetry; mo, months; yr, years.

**TABLE 3:** Pair‐wise correlations between selected socioemotional characteristics at 2 years and All psychopathology syndrome/categorical domains at 3 years.

	Social attention (2 yr)	Empathy (2 yr)	Social approach (2 yr)	Inhibition to novelty (2 yr)
Emotional reactivity (3 yr)	−0.320	−0.145	−0.004	0.048
	(0.025)*	(0.321)	(0.976)	(0.741)
Anxious/depressed (3 yr)	−0.208	−0.154	−0.021	0.106
	(0.152)	(0.290)	(0.884)	(0.468)
Somatic complaints (3 yr)	−0.231	0.062	−0.122	0.161
	(0.110)	(0.670)	(0.405)	(0.269)
Withdrawn (3 yr)	−0.496	−0.288	−0.124	0.105
	(<0.001)*	(0.045)	(0.396)	(0.471)
Sleep problems (3 yr)	−0.301	−0.092	<0.001	0.077
	(0.036)	(0.532)	(0.997)	(0.600)
Attention problems (3 yr)	−0.513	−0.216	−0.165	−0.310
	(<0.001)*	(0.135)	(0.256)	(0.030)
Aggressive behavior (3 yr)	−0.127	−0.058	0.055	−0.010
	(0.385)	(0.692)	(0.709)	(0.945)
Depressive problems (3 yr)	−0.347	−0.178	−0.063	0.084
	(0.015)*	(0.221)	(0.665)	(0.564)
Anxiety problems (3 yr)	−0.216	−0.022	0.047	0.222
	(0.136)	(0.879)	(0.747)	(0.125)
Autism spectrum problems (3 yr)	−0.432	−0.294	−0.146	0.096
	(0.002)*	(0.040)	(0.315)	(0.513)
Attention deficit/hyperactivity problems (3 yr)	−0.388 (0.006)*	−0.144 (0.324)	−0.063 (0.666)	−0.162 (0.266)
Oppositional defiant problems (3 yr)	−0.143 (0.326)	−0.064 (0.664)	0.103 (0.481)	0.051 (0.726)

*Note*: Exact effect sizes (first row) and *p*‐values (second row) are reported for each pairwise association. Associations that survive Benjamini–Hochberg FDR correction at *q* = 0.05 are marked with an asterisk.

Abbreviations: mo, months; yr, years.

### Structural equation model results

**FIGURE 2:**
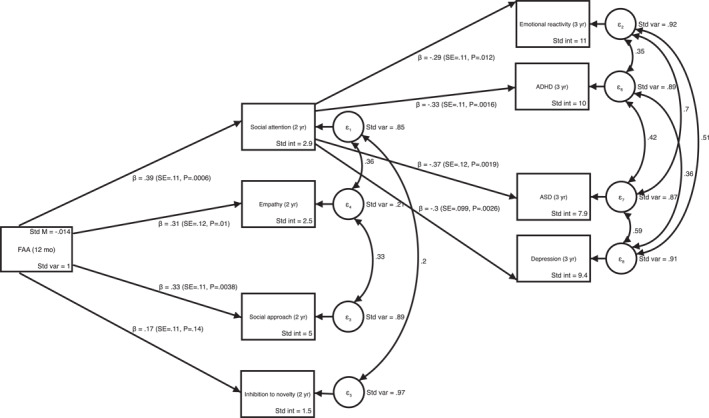
Structural equation model for frontal alpha asymmetry (12 months), child socioemotional characteristics (2 years), and child psychopathology symptoms (3 years). ADHD, Attention‐Deficit/Hyperactivity Disorder; ASD, Autism Spectrum Disorder; FAA, frontal alpha asymmetry; mo, months; yr, years.

A Structural Equation Model (SEM) was built to characterize prospective associations between FAA (assessed at multiple time points from 2 to 24 months), socioemotional characteristics at 2 years, and psychopathology symptoms/neurodevelopmental conditions at 3 years (Figure 2). The final model selected for having the best fit indices had a χ^2^ value of 28.141 and a *p*‐value of 0.081 (*p*‐values >0.05 considered to have adequate model fit). Root Mean Square Error of Approximation (RMSEA) values were 0.057 (values < 0.08 indicate acceptable fit), suggesting that the model approximates the population covariance structure with minor error. Model fit statistics were good to excellent, with CFI and TLI values at 0.953 and 0.912 respectively (values > 0.90 considered to have good relative fit compared to a baseline independent model). AIC and BIC values were 2306.6 and 2412.2 respectively and were lowest in the final model compared to other models fit.

In SEM modeling, we found that FAA at 12 months most saliently predicted socioemotional characteristics at 2 years and more distally psychopathology symptoms at 3 years. FAA at 12 months had direct effects with several socioemotional characteristics at 2 years of age including social attention (*β* = 0.39, *p* = 0.0006), empathy (*β* = 0.31, *p* = 0.01), and social approach (*β* = 0.33, *p* = 0.0038). All associations were positive, such that greater FAA values (i.e., greater relative LFA) were associated with greater social attention, social approach, and empathy levels at 2 years. FAA was not significantly associated with inhibition to novelty. Of the socioemotional characteristics modeled, only social attention had significant paths with psychopathology symptoms/neurodevelopmental conditions at 3 years. Specifically, higher levels of social attention were associated with lower levels of emotional reactivity (*β* = −0.29, *p* = 0.012) as well as with lower levels of categorical disorder symptoms including ADHD (*β* = −0.33, *p* = 0.0016), ASD (*β* = −0.37, *p* = 0.0019), and Depression (*β* = −0.30, *p* = 0.0026).

## DISCUSSION

In the current study, we sought to examine how early patterns of FAA, assessed longitudinally at multiple time points throughout the first 2 years of life, relate to various socioemotional characteristics at 2 years and psychopathology symptoms at 3 years. We found evidence that FAA at 12 months (greater relative LFA) predicted increased social attention, empathy, and social approach but not inhibition to novelty at 2 years. We also found that although FAA at 12 months predicted levels of social attention, empathy, and social approach at 2 years (and that each of these socioemotional characteristics covaried), only social attention served as a mediator for subsequent psychopathology symptoms/neurodevelopmental conditions. Specifically, lower levels of social attention were associated with greater emotional reactivity transdiagnostically (syndrome scale), and with ADHD, ASD, and Depression (DSM‐oriented scales). These findings suggest that social attention may be an important pathway through which FAA affects subsequent outcomes.

Of the time points assessed (2, 6, 9, 12, and 24 months), FAA asymmetry at 12 months was associated with socioemotional outcomes at 2 years including social attention, empathy, and social approach. Existing research suggests that 9–10 months marks a sensitive period during which infants are expecting environmental inputs that facilitate the synchronous coordination of social behavior, typically with an attachment figure (Feldman, 2015). This period typically marks synchronization of social gaze/attention (e.g., infants looking at caregivers when their name is called), and may explain why differences in FAA by 12 months of age may most strongly predict subsequent socioemotional responses (e.g., social attention, approach, and empathy) (Feldman, 2015). By the end of the second year of life, social synchronization tends to become more endogenously directed (i.e., internally motivated and goal‐directed; a product of voluntary orienting), perhaps explaining why FAA may be more weakly related to socioemotional characteristics at this time point (Falck‐Ytter et al., 2023). These findings add to the existing literature by suggesting that the end of the first year of life may be a time when FAA and socioemotional processes are most associated, although replication in additional samples is necessary.

Past work examining associations between FAA and socioemotional characteristics largely focused on the approach‐withdrawal hypothesis (Henriques & Davidson, 1991). Specifically, this hypothesis suggests that greater relative LFA is associated with approach behaviors and largely positive emotions, while RFA is associated with withdrawal behaviors and largely negative emotions (Henriques & Davidson, 1991). However, researchers have found that while patterns of LFA have been more consistently associated with behavioral approach responses in past literature, behavioral inhibition/withdrawal has been less consistently associated with RFA (Angus & Harmon‐Jones, 2016; Carver & White, 1994; Coan & Allen, 2003; Harmon‐Jones & Allen, 1997; Harmon‐Jones & Gable, 2018; Quaedflieg et al., 2015; Sutton & Davidson, 1997).

The current study adds to this literature by suggesting that while social approach responses/empathy (i.e., infants enjoying social contact, affection, smiling back, hugging others, and being interested in other children) may be linked with LFA, it may be differences in social attention that drive subsequent risk for psychopathology/neurodevelopmental conditions rather than behavior. Behavioral inhibition/withdrawal (e.g., taking a while to speak in unfamiliar situations, shyness with new adults/children, being quiet and less active in new situations) was not associated with FAA after accounting for social attention, approach, and empathy. Inconsistent findings for the association between FAA and behavioral inhibition/withdrawal in past work may reflect confounding by other socioemotional characteristics.

Diminished social attention (e.g., limited eye contract, differences in face processing, reduced joint attention) in those with neurodevelopmental disorders has historically been interpreted as a product of decreased social motivation or withdrawal (e.g., negative states of arousal to eye contact which then interfere with social attention). However, recent evidence suggests that reduced social approach in individuals with neurodevelopmental differences (e.g., ASD) may not be due to decreased social motivation and is not attributable to withdrawal/behavioral inhibition (Kim et al., 2015; Kylliainen et al., 2012; Moriuchi et al., 2017; Mundy & Bullen, 2021; Senju & Johnson, 2009). Instead, differences in social attention and subsequent behavioral responses (e.g., diminished social approach) may be due the product of differences in general motivational systems and/or executive functioning (e.g., heightened/diminished sensory processing, reduced attention shifting, reduced attention disengagement, differences in perspective‐taking) (Mundy & Bullen, 2021). The current work is in line with this emerging evidence, suggesting that FAA (at 12 months) may be linked with social attention (at 2 years; covarying with social approach/empathy but not inhibition to novelty) and consequently increase risk for emotional reactivity (at 3 years). This work also serves to start to reconcile inconsistencies in past brain‐behavioral research linking FAA to categorical disorders by suggesting that emotional reactivity may be a salient transdiagnostic symptom linked to FAA.

### Strengths and limitations

Some strengths of the current study include the use of a longitudinal design with multiple points of assessment across the first three years of life (2, 6, 9, 12, 24, and 36 months). Another strength is the consideration of various socioemotional characteristics and psychopathology symptoms/neurodevelopmental conditions (e.g., by symptom domains and DSM‐oriented scales), as well as the use of path modeling to better understand relationships between variables of interest. This study also focused on an understudied and underserved group, addressing a significant gap in existing research. Nonetheless, there are several limitations that should be addressed in future research.

First, future research with larger sample sizes will be important to determine whether the current findings are replicable in other samples and contexts. To examine a variety of longitudinal time points, socioemotional characteristics, and psychopathology/neurodevelopmental domains, the current study relied on exploratory preliminary analyses. Preliminary screening included many pairwise comparisons (i.e., FAA at 5 time points × 4 socioemotional characteristics; 4 socioemotional characteristics × 12 psychopathology/neurodevelopmental domains), which does increase the risk for type I errors. To mitigate this risk, paths in SEM models were selected using a combination of a priori decisions and the strength of effect sizes in preliminary analyses. Replication in future samples will be essential to determine whether this pattern of effects is consistent.

Additionally, while findings from the current study do align with some prior work and may clarify some of the inconsistencies in existing meta‐analytic work, additional replication in larger and independent samples will be necessary (Allen et al., 2018; Kolodziej et al., 2021; van der Vinne et al., 2017). Furthermore, incorporating multiple methods of assessment such as self‐report when developmentally appropriate, methods that involve naturalistic observation or real‐time monitoring of behavioral responses in various environmental contexts, and experimental paradigms would enhance our understanding of the pathways studied. Finally, future research is also needed to study these constructs (e.g., of FAA and psychopathology symptoms) from early childhood through later developmental time points in order to better understand how observed associations may shift over time.

### Conclusions

Taken together, existing research and the current study's findings suggest that differences in patterns for lateralization for FAA (12 months of age) may be most saliently linked with social attention at early developmental time points (2 years of age), and that social attention covaries significantly with other characteristics like empathy and social approach. This work suggests that patterns of activation for FAA may be more reflective of differences in attentional systems, especially those involved in social cognitions (e.g., social attention, perspective‐taking), than of specific behavioral responses like approach/withdrawal. Importantly, differences in FAA which subsequently influence social attention are associated with subsequent emotional reactivity (transdiagnostically across symptoms of ADHD, ASD, and depression).

The current pattern of findings for FAA and social attention as early predictors of transdiagnostic symptom risk highlight the need for future observational research and translational work clarifying early intervention targets. These findings, if replicated, could suggest that rather than targeting behavior alone (in line with behavioral approach/withdrawal frameworks through interventions like Behavioral Activation or Applied Behavior Analysis), or cognitive processes (in line with the current findings on social attention through interventions like Cognitive Restructuring, Mindfulness‐Based approaches, or Social Skills Training), other core treatment targets may be important to consider. As one example that would require further investigation, if the attentional differences that lead to decreased social attention and heightened emotional reactivity are the product of sensory processing differences as correlational work suggests (Ben‐Sasson et al., 2019; Dellapiazza et al., 2020; Huang et al., 2024; Li et al., 2023; Mimouni‐Bloch et al., 2018; Rani et al., 2023; Rogers & Ozonoff, 2005), interventions that facilitate optimum levels of arousal (e.g., Occupational Therapy to reduce sensory overload or increase sensory stimulation depending on the presenting concerns) may more effectively facilitate emotion regulation without increasing cognitive burden and further contributing to executive dysfunction and dysregulation. However, additional work will be needed to determine whether the current findings replicate in other samples, to further disentangle mechanisms, and test the effects of these treatment targets.

## AUTHOR CONTRIBUTIONS

**Viviane Valdes**: Conceptualization; writing—original draft; validation; visualization; writing—review and editing; formal analysis; project administration; data curation; supervision; investigation; methodology. **Niccola Lutri**: Writing—review and editing; project administration; formal analysis; validation. **Haerin Chung**: Writing—review and editing; methodology; validation. **Charles A. Nelson**: Funding acquisition; writing—review and editing; software; supervision; resources; project administration.

## CONFLICT OF INTEREST STATEMENT

The authors declare no conflicts of interest.

## ETHICAL CONSIDERATIONS

Parents or legal guardians provided written informed consent prior to the initiation of study activities; child assent was not sought as children were deemed too young to provide meaningful assent according to ethical guidelines. Study procedures were initially approved by the Boston Children's Hospital Institutional Review Board on 9/17/2015 and have been continually renewed through 9/26/2025 (IRB‐P00019083).

## Data Availability

The data that support the findings of this study are available from the corresponding author upon reasonable request.
